# Potential anti-*Pythium insidiosum* therapeutics identified through screening of agricultural fungicides

**DOI:** 10.1128/spectrum.01620-23

**Published:** 2024-01-05

**Authors:** Hanna Yolanda, Kedchin Jearawuttanakul, Warawuth Wannalo, Phongthon Kanjanasirirat, Suparerk Borwornpinyo, Thidarat Rujirawat, Penpan Payattikul, Weerayuth Kittichotirat, Duangdao Wichadakul, Theerapong Krajaejun

**Affiliations:** 1 Program in Translational Medicine, Faculty of Medicine, Ramathibodi Hospital, Mahidol University, Bangkok, Thailand; 2 Department of Parasitology, School of Medicine and Health Sciences, Atma Jaya Catholic University of Indonesia, Jakarta, Indonesia; 3 Excellent Center for Drug Discovery, Faculty of Science, Mahidol University, Bangkok, Thailand; 4 Department of Biotechnology, Faculty of Science, Mahidol University, Bangkok, Thailand; 5 Research Center, Faculty of Medicine, Ramathibodi Hospital, Mahidol University, Bangkok, Thailand; 6 Bioinformatics and Systems Biology Program, School of Bioresources and Technology and School of Information Technology, King Mongkut’s University of Technology Thonburi, Bangkhuntien, Bangkok, Thailand; 7 Systems Biology and Bioinformatics Research Group, Pilot Plant Development and Training Institute, King Mongkut’s University of Technology Thonburi, Bangkhuntien, Bangkok, Thailand; 8 Department of Computer Engineering, Faculty of Engineering, Chulalongkorn University, Bangkok, Thailand; 9 Center of Excellence in Systems Biology, Faculty of Medicine, Chulalongkorn University, Bangkok, Thailand; 10 Department of Pathology, Faculty of Medicine, Ramathibodi Hospital, Mahidol University, Bangkok, Thailand; University of Guelph, Guelph, Ontario, Canada

**Keywords:** pythiosis, *Pythium insidiosum*, oomycete, *in vitro *drug susceptibility, treatment

## Abstract

Pythiosis is a life-threatening infectious disease caused by the oomycete *Pythium insidiosum*. Clinical manifestations of pythiosis include an eye, blood vessel, skin, or gastrointestinal tract infection. Pythiosis has been increasingly reported worldwide, with an overall mortality rate of 28%. Radical surgery is required to save patients’ lives due to the limited efficacy of antimicrobial drugs. Effective medical treatments are urgently needed for pythiosis. This study aims to find anti-*P*. *insidiosum* agents by screening 17 agricultural fungicides that inhibit plant-pathogenic oomycetes and validating their efficacy and safety. Cyazofamid outperformed other fungicides as it can potently inhibit genetically diverse *P. insidiosum* isolates while exhibiting minimal cellular toxicities. The calculated therapeutic scores determined that the concentration of cyazofamid causing significant cellular toxicities was eight times greater than the concentration of the drug effectively inhibiting *P. insidiosum*. Furthermore, other studies showed that cyazofamid exhibits low-to-moderate toxicities in animals. The mechanism of cyazofamid action is likely the inhibition of cytochrome *b*, an essential component in ATP synthesis. Molecular docking and dynamic analyses depicted a stable binding of cyazofamid to the Qi site of the *P. insidiosum*’s cytochrome *b* orthologous protein. In conclusion, our search for an effective anti-*P*. *insidiosum* drug indicated that cyazofamid is a promising candidate for treating pythiosis. With its high efficacy and low toxicity, cyazofamid is a potential chemical for treating pythiosis, reducing the need for radical surgeries, and improving recovery rates. Our findings could pave the way for the development of new and effective treatments for pythiosis.

**IMPORTANCE:**

Pythiosis is a severe infection caused by *Pythium insidiosum*. The disease is prevalent in tropical/subtropical regions. This infectious condition is challenging to treat with antifungal drugs and often requires surgical removal of the infected tissue. Pythiosis can be fatal if not treated promptly. There is a need for a new treatment that effectively inhibits *P. insidiosum*. This study screened 17 agricultural fungicides that target plant-pathogenic oomycetes and found that cyazofamid was the most potent in inhibiting *P. insidiosum*. Cyazofamid showed low toxicity to mammalian cells and high affinity to the *P. insidiosum’s* cytochrome *b*, which is involved in energy production. Cyazofamid could be a promising candidate for the treatment of pythiosis, as it could reduce the need for surgery and improve the survival rate of patients. This study provides valuable insights into the biology and drug susceptibility of *P. insidiosum* and opens new avenues for developing effective therapies for pythiosis.

## INTRODUCTION

*Pythium insidiosum* is a filamentous oomycete microorganism that causes a severe infectious disease called pythiosis in humans and animals (1). Cases of pythiosis have been increasingly reported worldwide, especially in the past decade (2). The disease manifests in various clinical forms (1–3). Most human cases present as keratitis or arteritis of the extremity, while most affected animals present with cutaneous, subcutaneous, or gastrointestinal lesions (2). Many diagnostic methods have been developed to detect pythiosis early, which could lead to a better prognosis for affected patients (4). However, treatment of the disease remains a primary concern. The available therapeutic options, such as antimicrobial drugs (i.e., terbinafine, itraconazole, amphotericin B, linezolid, azithromycin, minocycline, and doxycycline), surgical intervention, and immunotherapy using *P. insidiosum* proteins, alone or in combination, show limited efficacy and result in high morbidity (such as the loss of infected organs like eye or leg) and overall mortality rate (13% for humans, 34% for animals, and 28% for all affected hosts) (2, 5–7). Responses to the treatment vary, which could be due to differences in host status, clinical stage, the organism’s virulence, and the effectiveness of the treatment. Notably, *P. insidiosum* lacks the proper target enzymes for conventional antifungal drugs, making the pathogen resistant to these drugs (8). Hence, finding a novel, and effective treatment for pythiosis is imperative.

Some non-conventional drugs/chemicals, such as natural compounds, bacterial metabolites, nanoparticles, antimicrobial peptides, photosensitizers, potassium iodide, triamcinolone, dimethyl sulfoxide (DMSO), ozone, prednisone, and mefenoxam, have been investigated for their inhibitory properties against *P. insidiosum* (5, 9–14). Of these, mefenoxam is an agricultural fungicide used to control plant-pathogenic microorganisms, such as *Phytophthora cinnamomi*, *Pythium myriotylum*, and *Pythium aphanidermatum*, that are phylogenetically related to *P. insidiosum* (15–17). An *in vitro* susceptibility test of mefenoxam against various *P. insidiosum* strains revealed a minimal inhibition concentration (MIC) of up to 10 µg/mL (14, 18). Another fungicide, pyraclostrobin, demonstrated an *in vitro* inhibitory effect against 21 *P*. *insidiosum* strains with MICs of up to 5 µg/mL (14). Toxicity evaluation using *Caenorhabditis elegans* showed that the worm could tolerate mefenoxam and pyraclostrobin (14). When mefenoxam was administered in combination with common antifungal drugs (itraconazole and terbinafine) and surgical intervention for up to 18 months, this treatment regimen cured six out of seven dogs with pythiosis (19, 20). Thus, agricultural fungicides are an exciting group of chemicals with potential applications in the fight against *P. insidiosum*.

Many fungicides have been used to control plant pathogens in agriculture. Our focus is on commercially available fungicides that inhibit plant-pathogenic oomycetes, but their properties against the human/animal-pathogenic oomycete *P. insidiosum* have yet to be evaluated. A literature review identified 17 fungicides (i.e., ametoctradin, fosetyl aluminium, pyraclostrobin, copper octanoate, cymoxanil, mancozeb, dimethomorph, oxathiapiprolin, fluopicolide, cyazofamid, fenamidone, mandipropamid, phosphoric acid, propamocarb hydrochloride, azoxystrobin, mefenoxam, and famoxadone) with growth-inhibitory activity against filamentous oomycetes, including *Phytophthora*, *Pseudoperonospora*, *Plasmopara*, and *Pythium* species (14, 15, 21–27). In this study, we evaluated the anti-*P*. *insidiosum* effect and cellular toxicity of these fungicides *in vitro* to find a potential chemical for effective pythiosis treatment. Additionally, using microbial genome data and molecular docking analyses, we examined the possible target protein and explored the mechanism of action of effective anti-*P*. *insidiosum* fungicides.

## MATERIALS AND METHODS

### Microorganisms

Twenty-one strains of *P. insidiosum* (Table 1) were recruited for the *in vitro* drug susceptibility testing against selected fungicides. The species identity of each organism was checked using PCR amplification and sequence analysis as described elsewhere (28, 29). The organisms were maintained and subcultured monthly on Sabouraud dextrose (SD) agar (1% peptone [Gibco Thermoﬁsher, MI, USA], 2% glucose [Himedia, Maharashtra, India], 1.2% agar [Difco BD, Le Pont de Claix, France], and distilled water; pH 7.2) until use.

**TABLE 1:** Twenty-one strains of *P. insidiosum* were used for *in vitro* susceptibility testing against three agricultural fungicides (i.e., cyazofamid, fenamidone, and fluopicolide)

No.	Strain ID	Source	Country of origin	Genotype (clade)	Concentration (µg/ml)					
					Cyazofamid		Fenamidone		Fluopicolide	
					MIC^*a*^	MCC^*b*^	MIC	MCC	MIC	MCC
1	Pi01	Equine	Costa Rica	I	≤1	4	32	128	8	16
2	Pi09^*c*^	Equine	Brazil	I	≤1	≤1	32	64	4	4
3	Pi10	Human	USA	I	≤1	≤1	64	64	4	8
4	Pi60	Equine	Brazil	I	≤1	≤1	≤1	4	≤1	≤1
5	Pi74	Dog	Thailand	I	≤1	16	64	128	≤1	2
6	Pi12	Human	Thailand	II	16	64	64	128	2	4
7	Pi23	Human	Thailand	II	2	16	64	64	4	4
8	Pi25	Human	Thailand	II	≤1	2	32	32	≤1	≤1
9	Pi35	Human	Thailand	II	16	32	64	128	2	4
10	Pi36	Equine	Australia	II	2	16	128	128	4	8
11	Pi37	Equine	New Guinea	II	8	16	128	128	4	4
12	Pi38	Human	India	II	≤1	4	64	64	8	16
13	Pi42	Environment	Thailand	II	≤1	16	16	64	2	2
14	Pi52^*c*^	Human	Thailand	II	16	16	64	128	≤1	≤1
15	Pi53	Equine	Thailand	II	≤1	4	32	64	2	2
16	Pi57	Human	Thailand	III	8	32	64	128	4	4
17	Pi75	Human	Thailand	III	≤ 1	2	16	64	≤1	≤1
18	Pi77	Environment	Thailand	III	8	32	128	128	2	2
19	Pi89	Environment	Thailand	III	16	16	64	128	4	4
20	Pi94	Human	Thailand	III	16	32	64	128	≤1	2
21	Pi50^*c*^	Human	USA	III	4	ND^*d*^	32	ND	4	ND
MIC and MCC ranges					≤1–16	≤1–64	≤1–128	4–128	≤1–8	≤1–16
MIC50^*e*^ and MCC50^*f*^					≤1	16	64	128	2	4
MIC90^*e*^ and MCC90^*f*^					16	32	128	128	4	8

^*a*^ Minimal inhibition concentration.

^*b*^ Minimal cidal concentration.

^*c*^ Strains recruited for the first-round *in vitro* drug susceptibility testing against selected fungicides.

^*d*^ ND, not done.

^*e*^ Drug concentration that completely inhibits at least 50% (MIC50) or 90% (MIC90) of the strains tested.

^*f*^ Drug concentration that kills a least 50% (MCC50) or 90% (MCC90) of the strains tested.

### Initial drug screening for chemical safety properties and toxicity

All recruited fungicides were initially screened for chemical safety properties by exploring PubChem (https://pubchem.ncbi.nlm.nih.gov/; accessed date: 1 June 2022). The fungicides that were corrosive, irritant, acute toxic, or health hazard were excluded from this study (Table 2).

**TABLE 2:** Seventeen anti-oomycete agricultural fungicides and their toxicity

Anti-oomycete fungicide (reference)	Toxicity^*a*^				Recruited for susceptibility and toxicity testing
	Corrosive	Irritant	Acute toxic	Health hazard	
Ametoctradin (21)	–^*b*^	Yes	–	–	–
Azoxystrobin (26)	–	–	Yes	–	–
Copper octanoate (23)	–	Yes	–	–	–
Cyazofamid (27)	–	–	–	–	Yes
Cymoxanil (22)	–	Yes	–	Yes	–
Dimethomorph (27)	–	–	–	–	Yes
Famoxadone (25)	–	–	–	Yes	–
Fenamidone (24)	–	–	–	–	Yes
Fluopicolide (15)	–	–	–	–	Yes
Fosetyl aluminum (22)	Yes	–	–	–	–
Mancozeb (26)	–	Yes	–	Yes	–
Mandipropamid (15)	–	–	–	–	Yes
Mefenoxam (14)	Yes	Yes	–	–	–
Oxathiapiprolin (15)	–	–	–	–	Yes
Phosphoric acid (15)	–	Yes	–	–	–
Propamocarb hydrochloride (21)	–	Yes	–	–	–
Pyraclostrobin (14)	–	–	Yes	Yes	–

^*a*^ Based on the laboratory chemical safety summary (LCSS) datasheet documented in the PubChem database (https://pubchem.ncbi.nlm.nih.gov).

^*b*^ – means no.

### *In vitro* drug susceptibility assays

Pure substances of six selected fungicides were purchased for *in vitro* susceptibility tests. These fungicides included mandipropamid (Sigma Aldrich, USA; ≥98.0% purity), fluopicolide (Sigma Aldrich, USA; ≥98.0% purity), cyazofamid (Sigma Aldrich, USA; ≥98.0% purity), fenamidone (Sigma Aldrich, USA; ≥98.0% purity), dimethomorph (Sigma Aldrich, USA; ≥98.0% purity), and oxathiapiprolin (Chem Service, USA, 98.7% purity). All chemicals were dissolved at the desired concentrations in 0.5% DMSO (Farmitalia Carlo Erba, Milano, Italy). Disulfiram (Unidrug Innovative Pharma Technologies, India; ≥98% purity), a repurposed drug that inhibits *P. insidiosum* (30), and 0.5% DMSO served as a positive and negative control agent, respectively, in the susceptibility test.

The radial growth assay was used to assess the antimicrobial activity of selected fungicides against *P. insidiosum* (9). An SD agar plug (4 mm in diameter) with actively-growing mycelium, also known as a hyphal plug, was excised from a 7-day-old *P. insidiosum* colony and served as the inoculum. The agar plug was placed on a 5-cm-SD agar plate containing a desired drug concentration before being incubated at 37°C for 2 days. Each *P. insidiosum* colony was measured twice for radial growth, and the results were averaged. The percent radial growth was calculated by comparing the average radial growth of the same *P. insidiosum* strain after exposure to an SD agar with and without a fungicide. MIC was defined as a fungicide concentration that exhibited undetectable growth on an agar plate. Hyphal plugs were taken from the SD agar plate containing the fungicide at MIC and higher concentrations and subjected to subculturing at 37°C for 2 days on a plain SD agar plate containing no drug to assess a minimal cidal concentration (MCC; the lowest drug concentration that can kill the organism). The terms MIC50 and MIC90 referred to the drug concentration completely inhibiting at least 50% and 90% of *P. insidiosum* strains tested. Likewise, the terms MCC50 and MCC90 are defined as the concentration that killed at least 50% and 90% of *P. insidiosum* strains tested, respectively.

The *in vitro* susceptibility test was performed in two rounds. The first round assessed, in triplicate, all six selected fungicides for their inhibitory activity against three *P. insidiosum* strains (Table 1). The second round tested, in duplicate, against 20 strains of the organism for only the fungicides that markedly exhibited anti-*P*. *insidiosum* effect (Table 1). The first-round assay employed SD agar plates containing serial drug concentrations ranging from 0.25 to 128 µg/mL, while the drug concentrations for the second-round assay ranged from 1 to 256 µg/mL.

### Assessment for drug toxicity

The selected fungicides were tested for cellular toxicity using the human corneal epithelium ATCC CRL-11135 (HCE-2 [50.B1]), human hepatocellular carcinoma ATCC HB-8065 (Hep G2), and human kidney proximal tubule ATCC CRL-2190 (HK-2) cell lines. The compositions for preparing the culture media of HCE-2 (50.B1), Hep G2, and HK-2 cell lines were summarized in Table 3. Propagation of only HCE-2 (50.B1) was required in a cell culture flask coated with a mixture of 0.01 mg/mL fibronectin (Invitrogen Thermo Fisher Scientific, Massachusetts, USA), 0.03 mg/mL bovine collagen type I (Invitrogen Thermo Fisher Scientific, Massachusetts, USA), and 0.01 mg/mL bovine serum albumin (HiMedia, Maharashtra, India). All cell types were incubated in a humidified incubator with 5% CO_2_ at 37°C.

**TABLE 3:** Compositions for preparing the culture of HCE-2 (50.B1), Hep G2, and HK-2 cells

Cell line	Medium composition		Manufacturer
HCE-2 (50.B1)	Basal medium	Keratinocyte serum-free medium	Invitrogen Thermo Fisher Scientific, Massachusetts, USA
	Supplements	0.05 mg/mL bovine pituitary extract	Invitrogen Thermo Fisher Scientific, Massachusetts, USA
		5 ng/mL epidermal growth factor	Invitrogen Thermo Fisher Scientific, Massachusetts, USA
		500 ng/mL hydrocortisone	HiMedia, Maharashtra, India
		0.005 mg/mL insulin	Lonza Walkersville, Maryland, USA
		100 units of penicillin-streptomycin	Gibco Thermo Fisher Scientific, New York, USA
Hep G2	Basal medium	Minimum essential medium	Gibco Thermo Fisher Scientific, New York, USA
	Supplements	10% fetal bovine serum	Merck, Darmstadt, Germany
		100 units of penicillin-streptomycin	Gibco Thermo Fisher Scientific, New York, USA
		1× non-essential amino acid	Gibco Thermo Fisher Scientific, New York, USA
HK-2	Basal medium	Low-glucose Dulbecco’s modified Eagle medium	Hyclone, Utah, USA
	Supplements	10% fetal bovine serum	Merck, Darmstadt, Germany
		100 units of penicillin-streptomycin	Gibco Thermo Fisher Scientific, New York, USA
		1× 4-(2-hydroxyethyl)-1-piperazineethanesulfonic acid (HEPES)	Hyclone, Utah, USA

The 3-(4,5-dimethylthiazol-2-yl)−2,5-diphenyl-2H-tetrazolium bromide (MTT) assay (31) was used to analyze the toxicity of the fungicides on the recruited cell lines, namely, HCE-2 (50.B1), Hep G2, and HK-2. Each cell type was seeded at a density of 5,000 cells/well and allowed to adhere for 24 h in a 96-microwell plate before treating with a fungicide at various concentrations (1–256 µg/mL). Doxorubicin (200 µM; Sigma Aldrich, USA) and DMSO (0.5%) were used as the positive and negative control, respectively. After 48-h incubation, the number of viable cells was determined by replacing the medium with 100 µL of 0.5 mg/mL MTT solution (Abcam, Cambridge, UK) and further incubating the cells for 3 h. The formed formazan crystals were solubilized with DMSO. The absorbance value was measured using a multimode microplate reader (ENVISION, PerkinElmer, USA) at 570 nm. The percent viability of the test cells exposed to a chemical of interest was calculated relative to those exposed to DMSO (negative control) (32). Each cell type was tested against each fungicide in triplicate for three independent experiments.

### Therapeutic score of anti-*P*. *insidiosum* fungicides

The therapeutic ratio for an anti-*P*. *insidiosum* fungicide is calculated by adapting the mathematical formula used to assess antibacterial drugs (33). The therapeutic score can be calculated through the ratio of drug toxicity (represented by toxic concentration 50 [TC50], which is a minimal drug concentration that reduces cell viability by at least 50%) to drug efficacy (represented by MIC90, which inhibits at least 90% of *P. insidiosum* strains tested).

### Target protein of cyazofamid, molecular docking, and molecular dynamics

Cytochrome *b* has been described as a cyazofamid target protein (34, 35). Its protein sequence was retrieved from the UniProt database (https://www.uniprot.org/; accessed date: 15 September 2022; UniProt ID: P00163), and BLAST searched for orthologous sequences of *P. insidiosum* deposited in the genome-derived proteome (36–38) and three susceptible hosts for pythiosis (i.e., human, horse, and dog) deposited in the NCBI database (https://blast.ncbi.nlm.nih.gov; accessed date: 5 April 2023). The cytochrome *b* orthologous sequence of *P. insidiosum* was compared with that of a human, a horse, and a dog for identity and similarity. All orthologous sequences were aligned using the Clustal Omega software (https://www.ebi.ac.uk/Tools/msa/clustalo/; accessed date: 12 April 2023) and viewed using the Jalview software (https://www.jalview.org/; accessed date: 12 April 2023).

The 3D structure of the *P. insidiosum*’s cytochrome *b* orthologous protein, namely, apocytochrome *b* (accession: YP_009167041.1), the putative target protein of cyazofamid, was predicted using AlphaFold2 (https://alphafold.ebi.ac.uk/; accessed date: 1 October 2022) (39). The structure of cyazofamid was obtained from PubChem (Compound identifier: 9862076; https://pubchem.ncbi.nlm.nih.gov/; accessed date: 1 October 2022). Both 3D structures of apocytochrome *b* and cyazofamid were converted to pdb format using Open Babel (40) and then to pdbqt format using AutoDockTools (41). The docking of the cyazofamid to a potential binding pocket (i.e., Qi binding site [42]) was performed using AutoDock Vina (43). The PyMOL program (Schrödinger, Inc., New York, USA) was used to visualize the 3D structure of the complex.

Regarding molecular dynamics simulation, hydrogens were added to cyazofamid using Avogadro software (44). ACPYPE-AnteChamber was used to generate the mol2 and topology files (45). The type of force field for a ligand (i.e., cyazofamid) was the general AMBER force field (GAFF), whereas for a protein (i.e., apocytochrome *b*) was AMBER ff14SB force field (46, 47). The TIP3P water model was used to simulate the solvent, and the steepest descent was chosen to solvate the system and for the energy minimization (48). The V-rescale was used for temperature coupling with a coupling constant of 0.1 ps (49). The electrostatic and van der Waals interactions were calculated using the particle-mesh Ewald algorithm and set 12 angstroms for the short-range van der Waals electrostatic (rcoulomb) cutoffs and neighbor list (rlist) (50). The LINCS algorithm was used to constrain all bond lengths and set the time step to 0.002 ps (51). The protein-ligand complex was equilibrated in NVT and NPT ensembles with 100 ps. The GROMACS 2020.4 was used to perform molecular dynamic (MD) simulations for 500 ns (52). The root mean square deviation of the cyazofamid’s heavy atom was measured. The binding free energy and per-residue decomposition were calculated using the molecular mechanics/Poisson-Boltzmann and surface area solvation (MM/PBSA) method by the gmx_MMPBSA program on the last 400 ns of simulations (53). The interaction entropy was also calculated by gmx_MMPBSA based on the method described in Duan et al. (54).

## RESULTS

### *In vitro* susceptibility testing of fungicides against *P. insidiosum*

**Fig 1:**
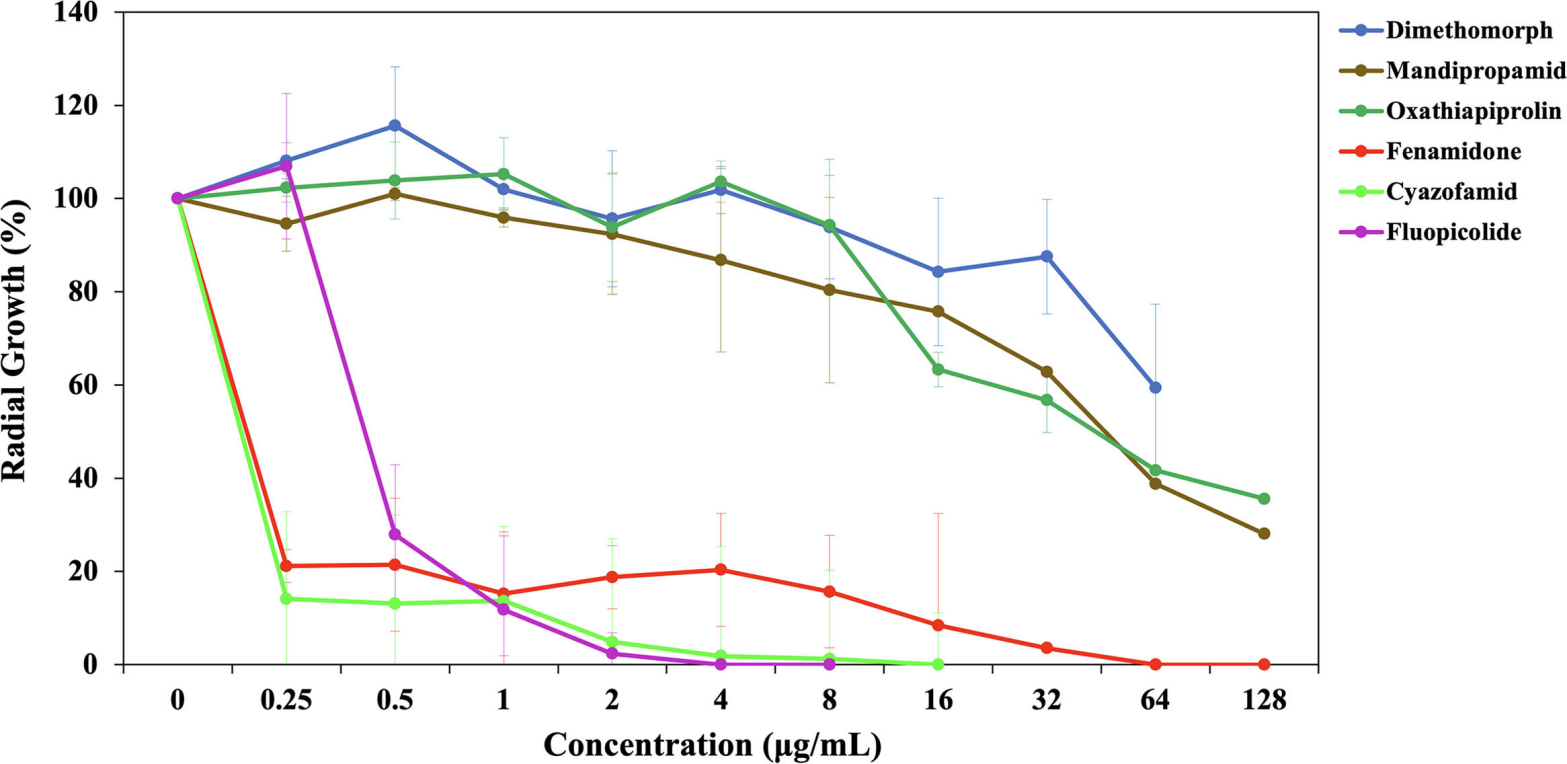
Anti-*P*. *insidiosum* activities of selected fungicides. Six anti-oomycete fungicides (i.e., fluopicolide, cyazofamid, fenamidone, mandipropamid, oxathiapiprolin, and dimethomorph) are recruited for an initial *in vitro* susceptibility analysis using radial growth assay, against three different isolates of *P. insidiosum* (i.e., Pi09, Pi52, Pi50).

**Fig 2:**
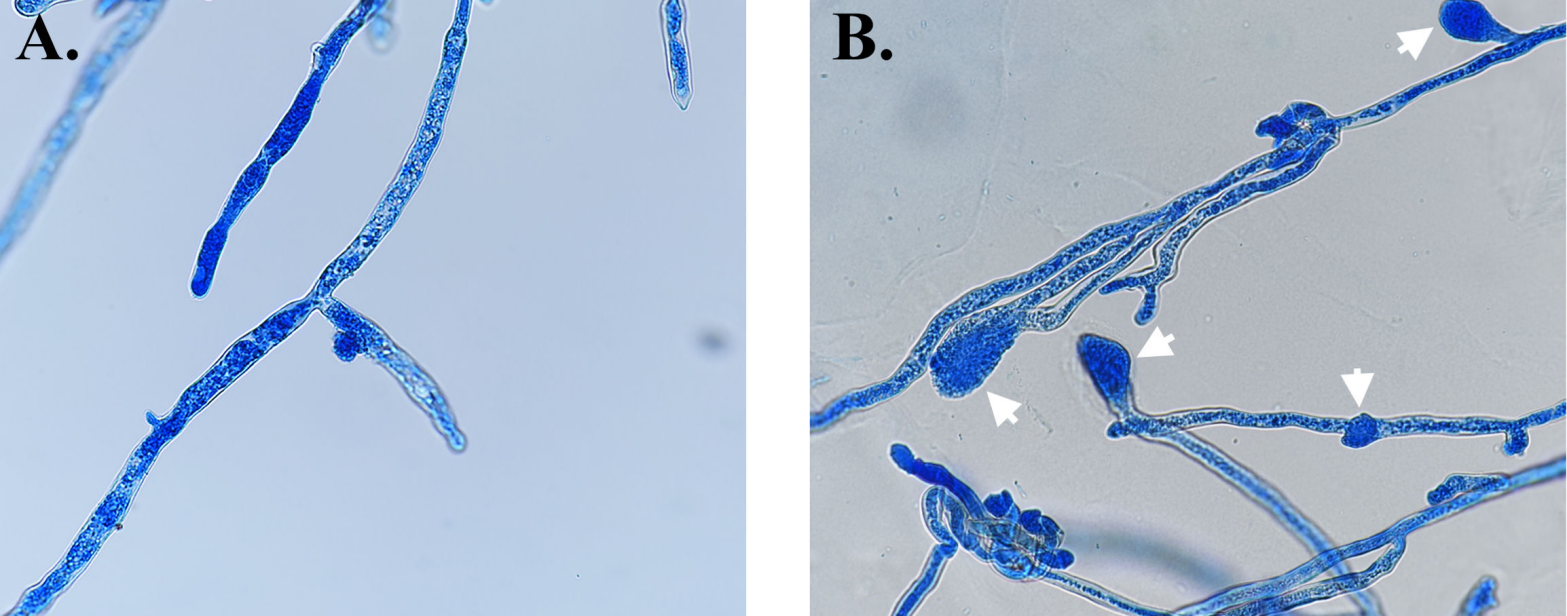
Microscopic features of *P. insidiosum* exposed to fungicides. As opposed to the no-drug control (**A**), in which microscopic appearance is intact, *P. insidiosum*, after exposure to a sublethal concentration of fluopicolide (i.e., 0.5 µg/mL), shows a balloon-like structure at some hyphal tips, as indicated by an arrow (**B**). The organism is stained with lactophenol cotton blue and examined under a light microscope at 400× magnification.

Six of 17 anti-oomycete fungicides (i.e., mandipropamid, fluopicolide, cyazofamid, fenamidone, dimethomorph, and oxathiapiprolin) were not categorized as corrosive, an irritant, acutely toxic, or as a health hazard chemicals (based on the PubChem database) were selected for the first-round *in vitro* susceptibility testing, using the radial growth assay (see Materials and Methods), against three *P. insidiosum* isolates (Table 2). Drug concentrations (prepared in twofold serial dilutions) used in the assay ranged from 0.25 to 128 µg/mL. Compared with the no-drug control, 0.5 µg/mL of fluopicolide, cyazofamid, or fenamidone can reduce the growths of *P. insidiosum* by at least 70% (Fig. 1). MICs of fluopicolide, cyazofamid, and fenamidone against the organisms were 4, 16, and 64 µg/mL, respectively, as no growth was observed at such drug concentrations (Fig. 1). In contrast, MICs of the other three fungicides (i.e., mandipropamid, dimethomorph, and oxathiapiprolin) were greater than 128 µg/mL (Fig. 1). Apart from the reduction in growth, the organisms exposed to fenamidone and cyazofamid, but not fluopicolide, showed decreased colony density compared with the no-drug control. The microscopic examination of *P. insidiosum* exposed to fluopicolide (0.5 µg/mL) demonstrated hyphae with a balloon-like structure at the tips that lacked perpendicular branching (Fig. 2). In contrast, the organisms exposed to cyazofamid (0.5 µg/mL) and fenamidone (32 µg/mL) showed no striking aberrant microscopic features.

Since only fluopicolide, cyazofamid, and fenamidone can completely inhibit *P. insidiosum* growth within the range of drug concentrations tested (Fig. 1), these chemicals were then subjected to the second round of *in vitro* susceptibility assessment against extended isolates (*n* = 20) from various hosts, geographic origins, and genotypes (Table 1). As a result, anti-*P*. *insidiosum* MICs and MCCs of each chemical were obtained and summarized in Table 1. In brief, fluopicolide had MICs ranging from ≤1 to 8 µg/mL (MIC50: 2 µg/mL; MIC90: 4 µg/mL) and MCCs ranging from ≤1 to 16 µg/mL (MCC50: 4 µg/mL; MCC90: 8 µg/mL). MICs and MCCs of cyazofamid appeared within ≤1–16 µg/mL (MIC50: ≤1 µg/mL; MIC90: 16 µg/mL) and ≤1–64 µg/mL (MCC50: 16 µg/mL; MCC90: 32 µg/mL), respectively. The ranges of MICs and MCCs of fenamidone were ≤1–128 µg/mL (MIC50: 64 µg/mL; MIC90: 128 µg/mL) and 4–128 µg/mL (MCC50 and MCC90: 128 µg/mL).

### Toxicity assessment and therapeutic scores of anti-*P*. *insidiosum* fungicides

**Fig 3:**
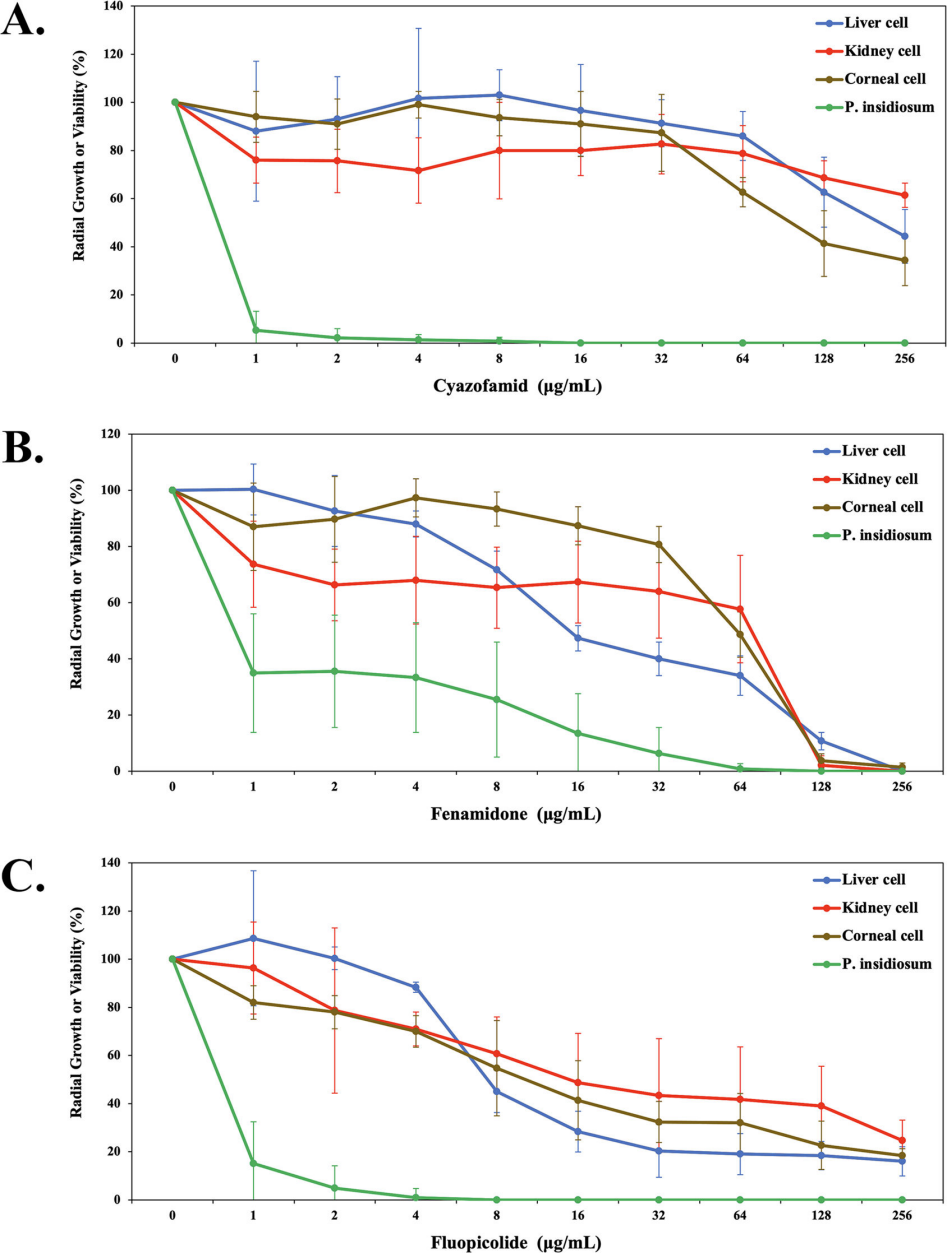
Assessment of anti-*P*. *insidiosum* activities and toxicity of three selected fungicides. Cyazofamid (**A**), fenamidone (**B**), and fluopicolide (**C**) are evaluated for antimicrobial effects against 20 isolates of *P. insidiosum*, using radial growth assay (reported as % radial growth), and for cellular toxicity against three different human cell lines (i.e., corneal epithelial cell [HCE-2 {50.B1}]), hepatocellular carcinoma cell (Hep G2), and kidney proximal tubule cell (HK-2), using MTT assay (reported as % cell viability).

Fluopicolide, cyazofamid, and fenamidone were also assessed for cellular toxicity using three different cell lines: corneal epithelial cell (HCE-2 [50.B1]), hepatocellular carcinoma cell (Hep G2), and kidney proximal tubule cell (HK-2). The viability of each cell type was recorded after exposure to twofold serial concentrations (range: 1–256 µg/mL) of each fungicide (Fig. 3). The cellular toxicity of each fungicide was dose-dependent, where the higher drug concentration led to lower cell viability. However, the degrees of cellular toxicity appeared different among fungicides. For example, fenamidone, at 128–256 µg/mL, dramatically caused an almost 100% reduction in the viability of all cell types. To a lesser extent, fluopicolide of at least 32 µg/mL diminished the cell viability by ~50%–80%. Cyazofamid showed relatively lower cellular toxicity, as it reduced the cell viability by less than 20% after exposure to this chemical up until 32 µg/mL. A higher concentration of cyazofamid (i.e., 64, 128, and 256 µg/mL) gradually decreased the cell viability by up to 70%. Concerning TC50, cyazofamid exhibited a relatively higher drug concentration range (128–>256 µg/mL) that reduced the viability of each cell line by at least 50%, followed by fenamidone (16–128 µg/mL) and fluopicolide (8–16 µg/mL), as summarized in Table 4.

**TABLE 4:** Therapeutic scores of cyazofamid, fluopicolide, and fenamidone against 20 isolates of *P. insidiosum*

Fungicide	MIC90^*a*^ (µg/mL)	TC50^*b*^ (µg/mL) and therapeutic score (TC50/MIC90)^*c*^	Type of cell line		
			Corneal epithelium (HCE-2 [50.B1])	Hepatocellular carcinoma (Hep G2)	Kidney proximal tubule (HK-2)
Cyazofamid	16	TC50	128	256	>256
		Therapeutic score	8.0	16.0	>16.0
Fenamidone	128	TC50	64	16	128
		Therapeutic score	0.5	0.1	1.0
Fluopicolide	4	TC50	16	8	16
		Therapeutic score	4.0	2.0	4.0

^*a*^ Minimal drug concentration that completely inhibits at least 90% (MIC90) of the strains tested.

^*b*^ Minimal drug concentration that reduces cell viability by at least 50% (TC50).

^*c*^ Can be calculated by dividing drug toxicity (TC50) by drug efficacy (MIC90).

The therapeutic score of fungicides measures a drug’s safety and compares the amount of a therapeutic agent that causes the therapeutic effect to the amount that causes toxicity. The therapeutic score can be calculated simply by dividing drug toxicity (TC50) by drug efficacy (MIC90). Regarding all types of cell lines tested, cyazofamid exhibited relatively higher therapeutic scores (range: 8.0–>16.0), followed by fluopicolide (range: 2.0–4.0) and fenamidone (range: 0.1–1.0) (Table 4). As it exhibited markedly high-therapeutic scores, cyazofamid was further investigated for its target protein and mechanism of action.

### Cytochrome *b* orthologous sequences of *P. insidiosum*, human, horse, and dog

Cyazofamid is known to bind with the Qi binding site of cytochrome *b* in the cytochrome *bc*1 complex (34, 42). The reference cytochrome *b* protein (UniProt ID: P00163; length: 385 amino acids) from *Saccharomyces cerevisiae* served as a query sequence for BLAST searching against the proteomes of *P. insidiosum* (Taxonomy Identifier [TAXID]: 114742) (34–38) and susceptible hosts to pythiosis, such as human (*Homo sapiens*; TAXID: 9606), horse (*Equus caballus*; TAXID: 9796), and dog (*Canis lupus familiaris*; TAXID: 9615). The best match for *P. insidiosum* was an apocytochrome *b* (accession: YP_009167041.1; *E*-value: 2e−147; identity: 56.0%; query coverage: 97%; length: 383 amino acids). The representative matches for the hosts were as follows: (i) accession AGZ77084.1 for the human (*E*-value: 6e−122; identity: 50.3%; query coverage: 97%; length: 380 amino acids); (ii) accession AFY15887.1 for the horse (*E*-value: 8e−133; identity: 52.4%; query coverage: 97%; length: 379 amino acids); and (iii) accession ABY80419.1 for the dog (*E*-value: 4e−136; identity: 52.1%; query coverage: 97%; length: 379 amino acids).

**Fig 4:**
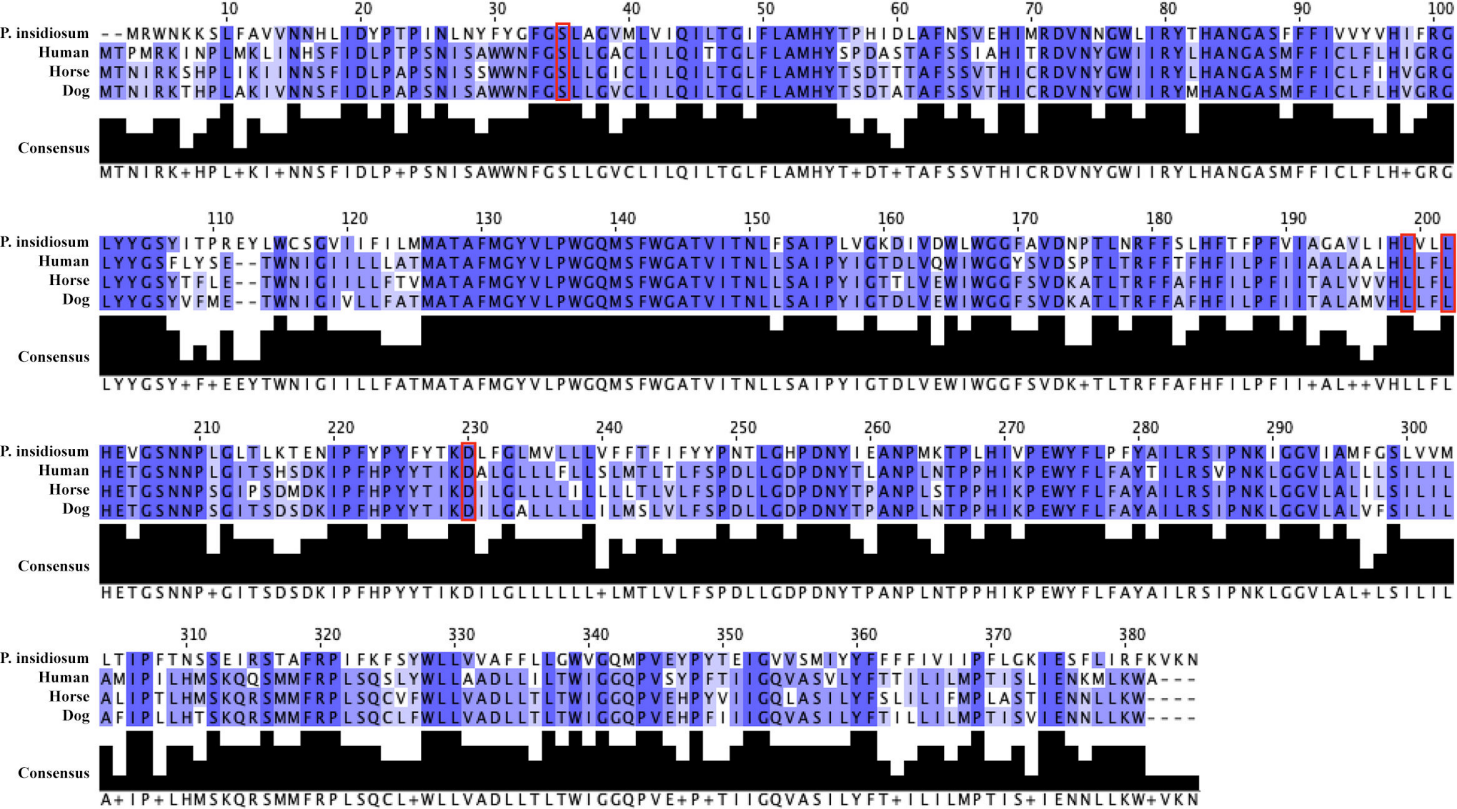
Sequence alignment of the cytochrome *b* orthologous proteins from *P. insidiosum*, human, horse, and dog. Red boxes show the predicted cyazofamid-binding sites of the *P. insidiosum* (i.e., Ser33, Leu197, Leu200, and Asp228) and host (i.e., Ser35, Leu197, Leu200, and Asp228) proteins.

The cytochrome *b* orthologous proteins from *P. insidiosum* and the hosts were compared. Sequence identities of cytochrome *b* orthologous proteins within the hosts (i.e., human, horse, and dog) ranged from 80.5% to 89.5%. However, the identities of the target protein sequence of *P. insidiosum* compared to that of hosts were markedly lower: 52.6% (human), 53.3% (horse), and 53.8% (dog). Predicted cyazofamid-binding sites of *P. insidiosum*’s apocytochrome *b* were at Ser33, Leu197, Leu200, and Asp228, which were similarly presented in the host proteins (i.e., Ser35, Leu197, Leu200, and Asp228). All cytochrome *b* orthologous sequences were aligned, and the cyazofamid-binding sites are shown in Fig. 4.

### Interaction of cyazofamid to a potential target protein in *P. insidiosum*

**Fig 5:**
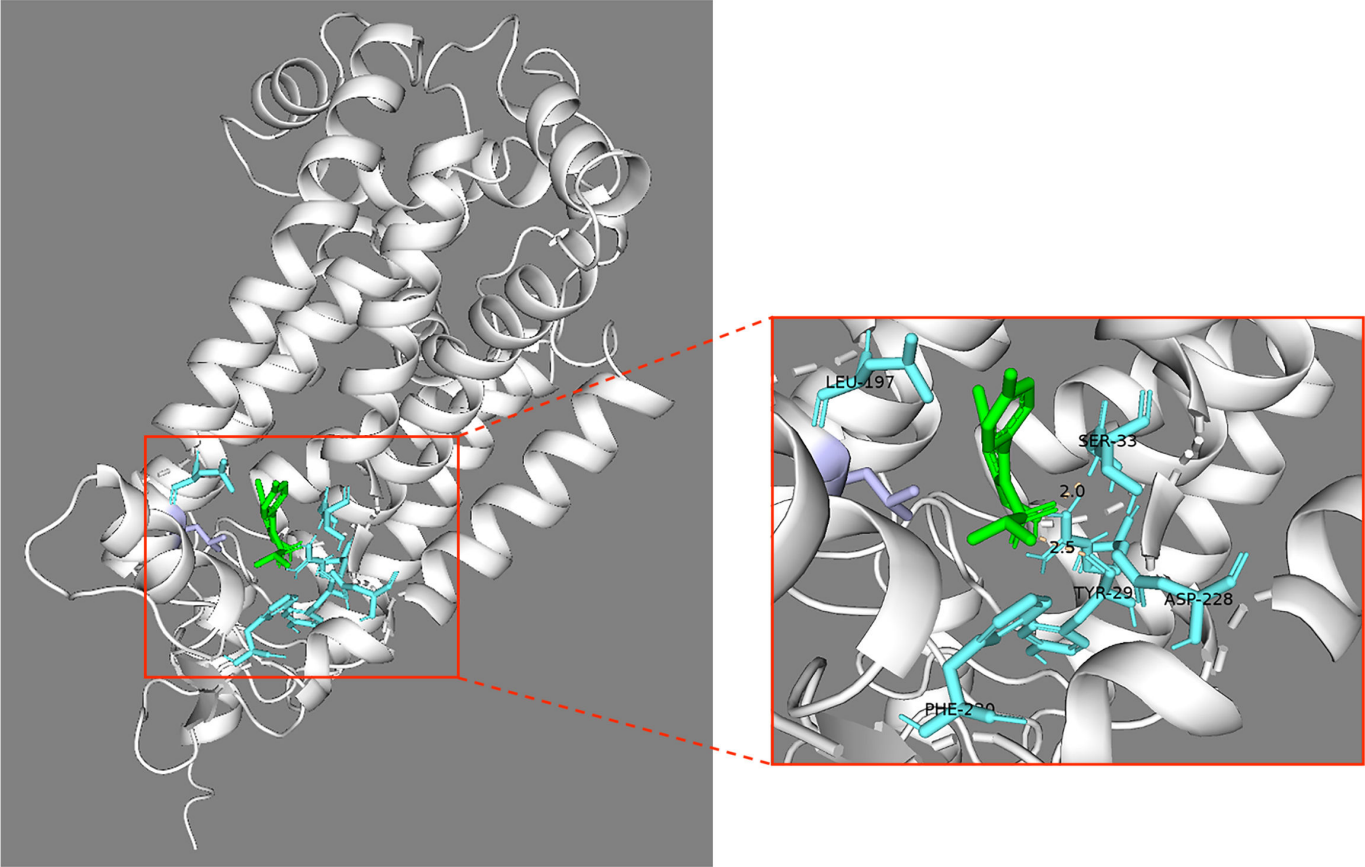
Docking analysis of cyazofamid and *P. insidiosum*’s apocytochrome *b*. Cyazofamid (green) can bind the 3D ribbon protein structure of the apocytochrome *b* (accession: YP_009167041.1), predicted by the AlphaFold2 software. The red box shows an enlarged area of the protein-ligand binding pocket of cyazofamid within apocytochrome *b*. The key binding amino acid residues include Ser33, Leu197, Leu200, and Asp228 (turquoise). The contacts are within 3.5 angstroms between cyazofamid and the target protein residues.

Docking analysis using the AutoDock Vina software (43) revealed that cyazofamid could bind to the 3D protein structure of a target protein, *P. insidiosum*’s apocytochrome *b* (accession: YP_009167041.1), predicted by the AlphaFold2 software (39), with a binding affinity score of −7.3 kcal/mol. Based on molecular dynamics simulation, the key binding amino acid residues between cyazofamid and the target protein residues were Ser33, Leu197, Leu200, and Asp228 (Fig. S1 and S2). Cyazofamid fitted in the binding pocket of the *P. insidiosum*’s apocytochrome *b*, surrounded by residues Tyr29, Gly32, Ser33, Arg98, Leu197, Phe220, and Asp228 within 3.5 angstroms, with two polar contacts to Tyr29 (2.5 angstroms) and Ser33 (2.3 angstroms) (Fig. 5). The Δ*G*_bind_ (indicating binding free energy to show how strong a chemical can bind its target protein) and −*T*Δ*S* (indicating interaction entropy when a chemical binds to a protein) of cyazofamid-apocytochrome *b* complex were −9.23 and 7.53 kcal/mol, respectively (see Table S1; Fig. S3 for other energetic components and Fig. S4 for the convergence of interaction entropy or −*T*Δ*S*).

## DISCUSSION

Agricultural fungicides (a group of chemicals with diverse structures and modes of action) have been used to fight against plant-pathogenic pathogens, including fungi and oomycetes (55). Their efficacy and safety should be carefully addressed when considering their potential for antimicrobial applications in medical and veterinary disciplines. Pythiosis is an infectious disease with high morbidity and mortality, partly due to the lack of effective antimicrobial drugs (2, 3, 56). The etiologic organism *P. insidiosum* resists conventional antifungal drugs (5, 8, 56). It is then justified to search for a new drug for treating pythiosis, and agricultural fungicides are an exciting group of chemicals that deserves exploration in this regard. Based on the literature review, we summarized a set of 17 fungicides that are potent for inhibiting plant-pathogenic oomycetes (Table 2). We investigated whether these chemicals exhibit antimicrobial activity against *P. insidiosum*, a prominent human- and animal-pathogenic oomycete. Eleven fungicides, including two previously-reported chemicals (mefenoxam and pyraclostrobin) with anti-*P*. *insidiosum* properties (14, 18), were excluded from our downstream assessment because of their documented toxicity to health (Table 2).

The remaining six fungicides were screened through several rounds of *in vitro* susceptibility analyses against diverse isolates of *P. insidiosum*. Only three, namely, fluopicolide, cyazofamid, and fenamidone, showed profound antimicrobial effects against the pathogen (Table 1; Fig. 1). Mechanisms of action for these anti-oomycete chemicals have been proposed by other investigators. For example, fenamidone and cyazofamid share an antimicrobial mode of action as they can affect the cytochrome *bc*1 complex, which is responsible for the electron transport of the respiratory chain in the mitochondria (34). These fungicides are proposed to bind a different site in the cytochrome *b* protein: the Qo site for fenamidone and the Qi site for cyazofamid (34, 57, 58). Fluopicolide may interfere with spectrin-like proteins, which involve the cytoskeleton structure of oomycetes (59, 60). The balloon-like structure observed at the hyphal tips of fluopicolide-exposed *P. insidiosum* (Fig. 2) could result from defected membrane cytoskeleton. It is worth noting that the other three fungicides (i.e., mandipropamid, oxathiapiproline, and dimethomorph) are effective against the oomycetes, primarily *Phytophthora* and *Pseudoperonospora* species (15, 27). However, we found these chemicals failed to markedly inhibit *P. insidiosum*, which is not a surprise since they also reportedly fail to control other *Pythium* species (61, 62), underlying the biological diversity among closely related oomycetes.

A potent antimicrobial agent could become useless if it possesses hazardous or toxic properties to patients. In this study, fluopicolide, fenamidone, and cyazofamid exhibited marked anti-*P*. *insidiosum* activities (Table 1; Fig. 1). We took a step further to assess their toxicity against available human cell lines originating from the liver (Hep G2), kidney (HK-2), and cornea (HCE-2 [50.B1]). Hep G2 and HK-2 are commonly used cells in drug toxicity studies, as they are derived from the major organs influencing drug metabolism, distribution, and clearance (63, 64). Hep G2 has a high-proliferation capability and retains certain features of the differentiated hepatocytes (63, 65). HK-2 is an immortalized proximal tubular epithelium of the kidney, and its function is to maintain fluid, electrolyte, and nutrient homeostasis (66, 67). We also included the corneal epithelium HCE-2 (50.B1) because the ocular infection is the most prevalent form of human pythiosis (1, 2). A good fungicide should be non-toxic chemicals or toxic only at high concentrations to host cells while preserving a potent antimicrobial property at low concentrations. This study used a calculated therapeutic score (also known as selectivity index or safety ratio [33, 68]) to determine how many times greater the concentration of a drug causing significant toxicity (TC50) in various cell types (i.e., Hep G2, HK-2, and HCE-2 [50.B1]) is than the concentration of a drug effectively inhibiting genetically diverse isolates of *P. insidiosum* (MIC90), as shown in Fig. 3; Table 4.

Fenamidone had therapeutic scores equal to or less than 1, indicating its effective anti-*P*. *insidiosum* concentration already led to significant cellular toxicity. Fluopicolide showed slightly improved therapeutic scores (range: 2–4) although its expected safety to the host cells was marginal. Cyazofamid, on the other hand, exhibited remarkably higher therapeutic scores, ranging from 8 to greater than 16, compared with the other fungicides tested, indicating that cyazofamid’s toxic concentration was much higher than its effective antimicrobial concentration and, thus, endorsing, to some extent, its safety use. In animals (i.e., rats, rabbits, guinea pigs, and dogs), cyazofamid shows low to moderate toxicities with no evidence of mutagenic, carcinogenic, neurotoxic, or developmental toxic properties (69, 70). Cyazofamid has low-intestinal permeability and poor interaction with human drug transporters, which could lead to minimal systemic toxicity and adverse effects (71). Among the 17 anti-oomycete fungicides recruited (Table 2), cyazofamid was outstanding for its efficacy against pathogen growth and limited cellular toxicity, making it a promising chemical for treating pythiosis.

Cyazofamid can interfere with the cytochrome *bc*1 complex (comprising three subunits: cytochrome *b*, cytochrome *c*1, and iron-sulfur protein), which is present at the inner mitochondrial membrane (34, 42, 58). As an essential process for life, the cytochrome *bc*1 complex involves the respiratory chain electron transfer to generate proton gradient and membrane potential as a part of ATP synthesis (42, 58). This process is called Q-cycle that requires two quinone-binding sites in cytochrome *b*: the quinol oxidation (Qo) site and the quinone reduction (Qi) site (42, 58). Cyazofamid is known to inhibit the Qi site (42). Besides cyazofamid, some antimicrobial drugs can also act by binding the Qo (i.e., atovaquone, hydroxynapthoquinone, and azoxystrobin) or Qi (i.e., pyridones, ametoctradin, and antimycin) site (34, 35, 42, 58). Among them, atovaquone is used to treat patients with pneumonia caused by the fungus *Pneumocystis jiroveci* (72), suggesting interference with cytochrome *b* is applicable in medicine. We attempted to gain insight into the mechanism of cyazofamid action against *P. insidiosum*. As such, we identified apocytochrome *b* (an orthologous cytochrome *b* and a possible target protein of cyazofamid) from the *P. insidiosum* proteome (35–38, 42, 58). We employed molecular docking and molecular dynamics simulation to predict if cyazofamid could bind to the apocytochrome *b* of *P. insidiosum*. The results showed that cyazofamid could potentially bind to the apocytochrome *b* with the same binding pocket of cytochrome *b* described in the bovine (42) but with a better (lower) ∆*G*bind of −9.23 kcal/mol in *P. insidiosum* compared with −3.3 kcal/mol in bovine based on the MM/PBSA method. Nevertheless, the apocytochrome *b*’s residues forming the H-bonds with cyazofamid were Ser33 and Tyr29, whereas Li et al. reported the H-bond with only between the residue Asp228 of bovine’s cytochrome *b* and cyazofamid (42).

The cytochrome *b* orthologous proteins were identified in *P. insidiosum* and its disease-susceptible hosts (i.e., humans, dogs, and horses). Besides, the predicted cyazofamid-binding sites of *P. insidiosum*’s apocytochrome *b* (i.e., Ser33, Leu197, Leu200, and Asp228) were similar to the host proteins (i.e., Ser35, Leu197, Leu200, and Asp228) (Fig. 4 and 5). Thus, it is conceivable that cyazofamid might show comparable cell inhibition in the pathogen and the hosts. However, the high therapeutic scores of cyazofamid (range: 8–16) indicated that this chemical selectively inhibited *P. insidiosum*, as opposed to the host cells (i.e., human, horse, and dog) (Fig. 3; Table 4). In line with our findings, Mitani et al. reported that cyazofamid specifically diminishes cytochrome *bc*1 complex activity in *Pythium spinosum* but not in other cells or organisms tested, such as *Botrytis cinerea*, *S. cerevisiae*, rat liver, and potato tuber (73). Differences in the sequence identities of the cytochrome *b* orthologous proteins (*P. insidiosum* vs hosts: 52.6%–53.8%; among hosts: 80.5%–89.5%; Fig. 4) might be responsible for the preferable inhibitory effect of cyazofamid against various organisms.

In conclusion, we have discovered that cyazofamid is a highly effective fungicide that can consistently and potently inhibit genetically diverse isolates of *P. insidiosum* while exhibiting minimal toxicities against various cell types. In fact, cyazofamid outperformed other fungicides that were screened for anti-*P*. *insidiosum* efficacy. The calculated therapeutic scores determined that the concentration of cyazofamid causing significant cellular toxicities is 8–16 times greater than the concentration of the drug effectively inhibiting *P. insidiosum*. Furthermore, other studies have shown that cyazofamid exhibits low to moderate toxicities in animals. The mechanism of cyazofamid action is likely the inhibition of cytochrome *b*, an essential component in respiratory chain electron transfer and ATP synthesis. Molecular docking and dynamic analyses have depicted a stable binding of cyazofamid to the Qi site of the *P. insidiosum* apocytochrome *b*, an orthologous protein of cytochrome *b*. Our search for an effective anti-*P*. *insidiosum* drug has indicated that cyazofamid is a promising candidate for treating pythiosis. With its high efficacy and low toxicity, cyazofamid is a potential chemical for treating pythiosis, reducing the need for radical surgeries, and improving recovery rates. Our findings could pave the way for further investigations (i.e., animal studies and clinical trials) for the clinical effectiveness of cyazofamid and the development of new and effective treatments for pythiosis.

## SUPPLEMENTAL MATERIAL

Supplemental figures and tables spectrum.01620-23-s0001.pdf Figures S1 to S4 and Table S1.

ASM does not own the copyrights to Supplemental Material that may be linked to, or accessed through, an article. The authors have granted ASM a non-exclusive, world-wide license to publish the Supplemental Material files. Please contact the corresponding author directly for reuse.
