# Supplementary material for: Potential anti-Pythium insidiosum therapeutics identified through screening of agricultural fungicides
Source: Microbiol Spectr. 2024 Jan 5;12(2):e01620-23. doi: 10.1128/spectrum.01620-23 (PMC10846074; doi:10.1128/spectrum.01620-23)
Supplement: Supplemental figures and tables — Figures S1 to S4 and Table S1. [file spectrum.01620-23-s0001.pdf]

SUPPLEMENTARY DATA

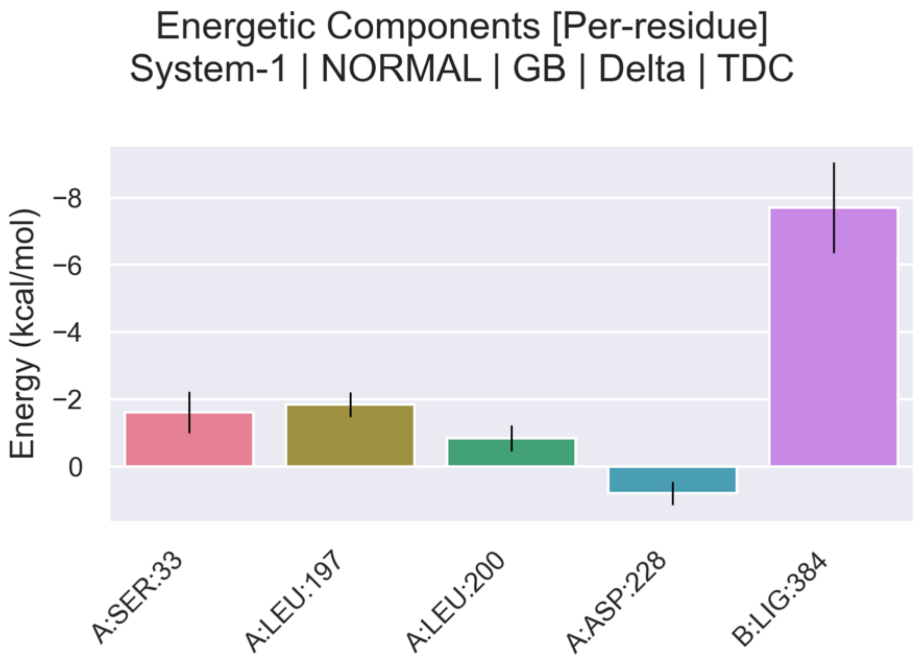

Supplementary Figure S1. The overall energetic components based on per-residue decomposition analysis at the cyazofamid-binding pocket of *P. insidiosum*'s apocytochrome b (Accession: YP_009167041.1).

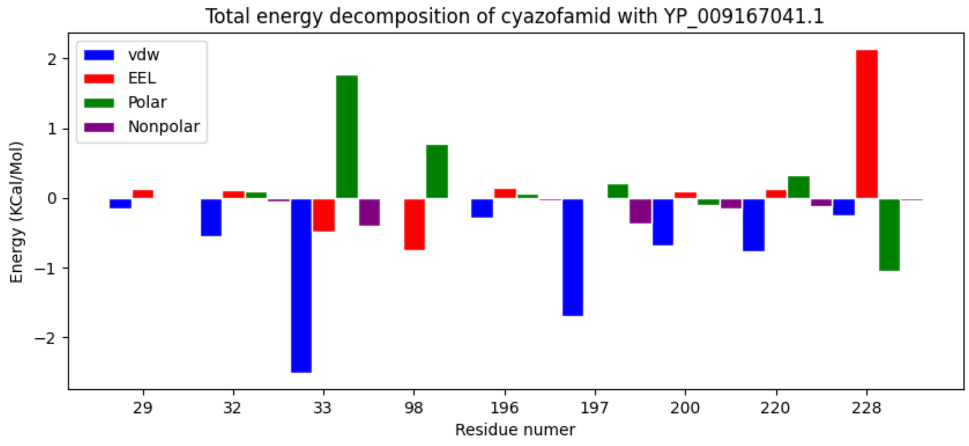

Supplementary Figure S2. The detailed energetic components based on per-residue decomposition analysis at the cyazofamid-binding pocket of *P. insidiosum*'s apocytochrome b (Accession: YP_009167041.1).

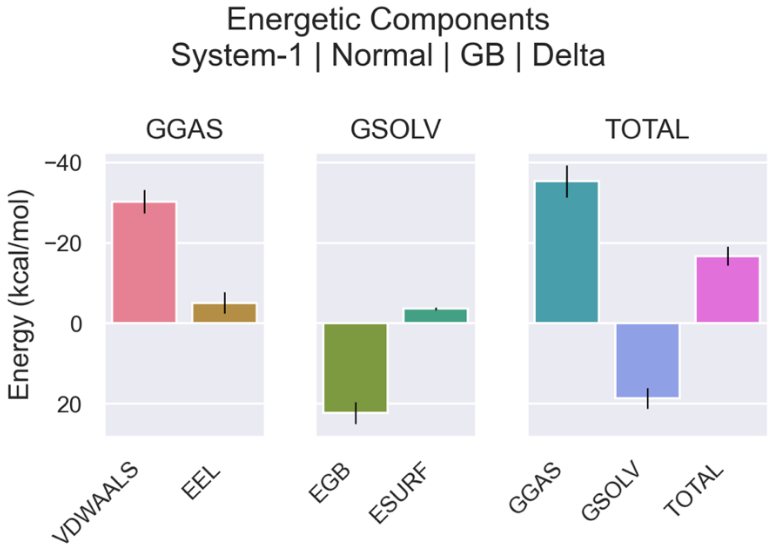

Supplementary Figure S3. The energetic components of the cyazofamid-apocytochrome b complex calculated by the gmx_MMPBSA program.

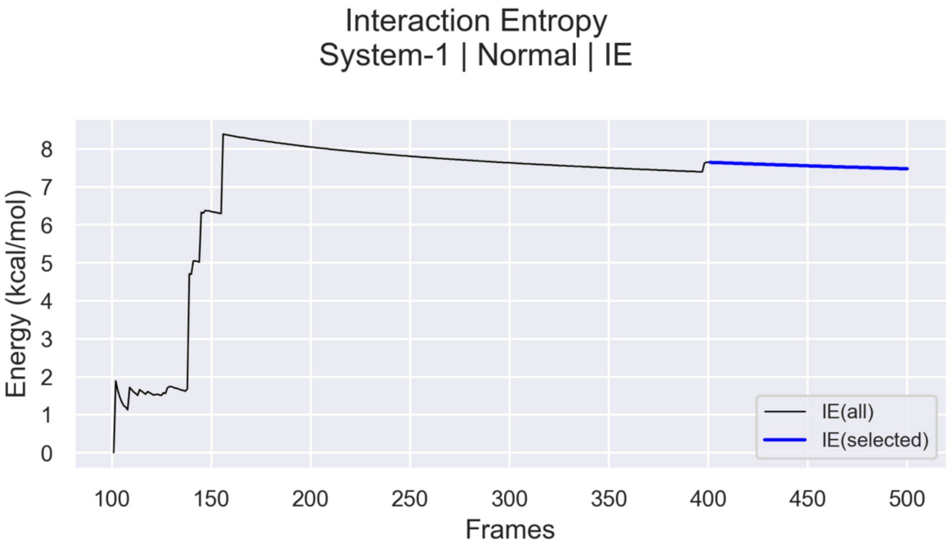

Supplementary Figure S4. The interaction entropy (-TΔS) of the cyazofamid-apocytochrome b complex calculated by the gmx_MMPBSA program.

Supplementary Table S1. The binding free energy (ΔG_{bind}) and its energetic components of the cyazofamid-apocytochrome b complex calculated by the gmx_MMPBSA program.

**Energetic components**	**Average/STD**
ΔEEL	-5.10 ± 2.65
ΔVDWAALS	-30.24 ± 2.91
ΔE_{MM} (GGAS)	-35.33 ± 3.99
ΔEGB	22.24 ± 2.72
ΔESURF	-3.67 ± 0.30
ΔTOTAL	-16.77 ± 2.43
-TΔS	7.53 ± 0.07
ΔG_{bind}	-9.23 ± 2.43
